# Service users’ involvement in the development of individual Cognitive Stimulation Therapy (iCST) for dementia: a qualitative study

**DOI:** 10.1186/s12877-015-0004-5

**Published:** 2015-02-06

**Authors:** Lauren A Yates, Martin Orrell, Aimee Spector, Vasiliki Orgeta

**Affiliations:** North East London Foundation Trust, London, UK; Division of Psychiatry, University College London, London, UK; Research Department of Clinical, Educational and Health Psychology, University College London, London, UK

**Keywords:** Cognitive stimulation therapy, Dementia, Focus groups, Individual cognitive stimulation therapy, Individual interviews

## Abstract

**Background:**

Individual Cognitive Stimulation Therapy (iCST) is a one to one, carer led psychosocial intervention for people with dementia, adapted from group Cognitive Stimulation Therapy (CST). It is increasingly recognised that involving service users in research is key to developing interventions and treatments that successfully address their needs. This study describes the contribution of people with dementia and carers during the development phase of the intervention and materials.

**Methods:**

Twenty-eight people with dementia and 24 carers were consulted in a series of six focus groups and 10 interviews. The purpose of this study was to gain insight into perceptions of mental stimulation from the point of view of carers and people with dementia, to ensure the materials are easy to use, clear, and appropriately tailored to the needs of people with dementia and their carers, and to assess the feasibility of the intervention.

**Results:**

The importance of mental stimulation was emphasized by carers and people with dementia. People with dementia saw activities as a way of ‘keeping up to date’ and spending time in a meaningful way. Carers reported benefits such as improved quality of life, mood and memory. The concept of iCST was well received, and both carers and people with dementia responded positively to the first drafts of materials. Feasibility issues, such as finding time to do sessions, were identified.

**Conclusion:**

The feedback from the focus groups and interviews will be used to further develop and refine the iCST programme materials in preparation for a field testing phase prior to a large scale randomized controlled trial (RCT).

**Trial registration:**

ISRCTN65945963. Date of registration: 05/05/2010.

## Background

Providing care for people with dementia is a great challenge for health and social care systems across the globe [1]. The number of people affected by the disease, coupled with escalating costs to the economy, highlights the pressing need to improve the quality of care and services available [2]. The development of user-friendly, clinically effective therapeutic interventions delivered by carers could improve cognition and quality of life for people with dementia, and help carers be involved in worthwhile and enjoyable activities. The Department of Health, the National Health Service (NHS) Executive, research charities and funding bodies suggest that the key to developing interventions and treatments that successfully address the needs of service users, and produce results that will impact clinical practice is to involve them in clinical research [3].

Group CST is an evidence-based psychosocial intervention for people with dementia [4]. Studies evaluating the effectiveness of CST consistently report that CST can improve cognition and quality of life for people with dementia [5]. Clinically and cost-effective [6], CST is recommended by the National Institute of Clinical Excellence (NICE) [7], the NHS Institute for Innovations and Improvements [8]. An extended programme of maintenance CST (14 sessions over 7 weeks plus a further 24 weekly sessions), developed and evaluated in a large scale randomized controlled trial, showed improvements in quality of life for people with dementia at 6 month follow up and further cognitive benefits for people on cholinesterase inhibitors [9]. CST groups are being integrated into NHS services nationally, and further research is underway into the implementation of CST and maintenance CST in practice [10]. However, for those unable to participate in groups due to local service constraints, personal preference, or health or mobility issues, an individualised carer-led version of CST would be beneficial.

The current study is part of a Health Technology Assessment (HTA) funded randomized clinical trial aiming to develop and evaluate iCST for dementia. With guidance from experts in dementia, including service users, healthcare professionals and academics, the CST and maintenance CST programmes were adapted to create a 25 week long structured programme of mentally stimulating activities designed to be delivered at home by family carers. A set of materials including a manual, activity workbook and toolkit were created for the programme.

The principles of the Medical Research Council (MRC) framework [11] were applied in the development of the iCST intervention and materials. This study describes the consultations with carers and people with dementia, which were carried out in accordance with Phase 1 (modelling) of the MRC guidelines. The purpose of the modelling phase was to gain insight into perceptions of mental stimulation from the point of view of carers and people with dementia, to ensure the intervention materials were easy to use, clear, and appropriately tailored to the needs of people with dementia and their carers, and to assess the feasibility of the programme.

## Methods

### Design

Semi-structured interviews and focus groups were selected as complimentary qualitative methods to assess the feasibility of the iCST programme, and the quality of the first draft of the materials produced. People with dementia and carers were consulted separately as well as collaboratively, to ensure both parties could express their opinions and outline their preferences for the programme, which may be disparate according to their role and needs [12,13]. A discussion guide was developed prior to the focus groups and interviews. The guide included open questions designed to promote discussion around mentally stimulating activities in general terms, and more focused questions that invited specific responses to the iCST materials provided at the sessions (see Table 1). Discussion of practical issues (eg: ‘How long should sessions last?’) constituted a key part of the guide produced. The guide was altered slightly for the groups and interviews with people with dementia, as these sessions were intended to be more focused on trying the activities than the practicalities of delivering the programme.

**Table 1:** **Discussion guide themes**

**Themes**	**Focus points**	**Group**			**Interview**	
		**Carer**	**PwD**	**Combined**	**Carer**	**PwD**
**Mental stimulation**						
	Importance of mental stimulation	X	X	X	X	X
	Mentally stimulating activities	X	X	X	X	X
**iCST manual**						
*Content*	Spelling/grammar	X		X	X	
	Appropriate language	X		X	X	
	Adequate explanations of terminology and concepts	X		X	X	
	Ideas for additional information	X		X	X	
*Layout*	Size of text & images	X		X	X	
	Clarity of layout	X		X	X	
	Images	X		X	X	
	Format (eg: ring bound)	X		X	X	
*General*	Positive comments	X		X	X	
	Negative comments	X		X	X	
	Ease of use	X		X	X	
**iCST activity workbook**						
*Content*	Clarity of instructions	X		X	X	
	Activities	X		X	X	
*Layout*	Format (eg: ring bound)	X		X	X	
	Clarity of layout	X		X	X	
	Images	X		X	X	
**Activity**						
	Difficulty of activity completed in session		X	X		X
	Level of stimulation/engagement		X	X		X
	Level of enjoyment		X	X		X
	Ideas to improve activity		X	X		X
**Feasibility**						
	Acceptability of delivering/receiving a home based programme	X	X	X	X	X
	Acceptability of programme schedule (eg: 3, 30 min sessions per week)	X		X	X	
	Acceptability of providing own materials	X		X	X	
	Anticipated practical difficulties	X		X	X	
	Support needed	X		X	X	
	Acceptability of telephone support and visits	X		X	X	
	Group training vs. one to one home based training	X		X	X	

### Sample

The research team worked in partnership with voluntary sector organizations (Carers of Lewisham, Jewish Care, Crossroads, Dementia UK, Staywell), memory services and a day centre unit in North East London Foundation Trust (NELFT), and a local authority organization (Living Well Resource Centre, Redbridge) to recruit participants. The organizations recruited for focus groups if they were able to provide a venue, and interviews if they did not have the facility to host a group.

The organizations made initial contact with carers and people with dementia who were suitable and interested in the research activities, typically approaching them during support groups, or during memory clinic appointments. The research team then contacted consenting dyads to confirm their eligibility and determine their availability for a local focus group, or negotiate a convenient time for an interview. People were eligible to participate if they had a diagnosis of mild to moderate dementia (meeting the Diagnostic and Statistical Manual of Mental Disorders 4^th^ Edition [DSM-IV] criteria & score of 10 or above on the Mini Mental State Examination [MMSE]) [14,15], were able to communicate and understand communication, and able to provide informed consent to take part in a discussion group or interview, and were living in the community. Carers were eligible to participate if they were currently caring for a person with dementia.

### Procedure

#### Focus groups

Of the nine groups planned, six were conducted; two with carers, three with people with dementia, and one with both carers and people with dementia. Each group was attended by two members of the research team, one of whom took on the role as facilitator and led the group discussion, and the other observed the group and made notes to supplement the audio data collected. The discussions were conducted in a semi-structured style guided by a series of pre determined focus points and questions (Table 1). During the session, participants were invited to appraise and interact with a selection of sample materials from the programme including manuals, activity workbooks and toolkit items. Each session lasted approximately 90 minutes in total.

### Individual interviews

Ten interviews were planned and conducted. People with dementia and their carers were interviewed separately. The interview with the person with dementia was conducted first to allow the carer time to appraise a set of sample materials. Typically each interview lasted 30–45 minutes. Interviews with people with dementia involved completing two iCST activities, specific feedback about their enjoyment and comprehension of the activities, and a general discussion about perceptions of, and needs for a home based programme of mentally stimulating activities. The main aims of the carer interviews were to identify any practical issues that might affect the delivery of the programme, and to gather data about the quality and appropriateness of the activities and manuals, which would inform the development of further drafts of the materials (Table 1).

### Ethical considerations

Information sheets for carers and people with dementia were approved by the Multi-centre Research Ethics Committee (ref no.10/H0701/71). All participants received the information sheets a minimum of 24 hours before the scheduled research activities in accordance with Good Clinical Practice guidelines. At the beginning of each session, researchers reiterated information about the procedure and aims of the groups and interviews and offered participants the opportunity to raise any queries. People with dementia were in the mild to moderate stages of dementia, and thus were deemed able to provide informed consent for participation.

Written consent was obtained on the day of the research activity. Continuing assent was established by informing participants that they were free to leave the group or terminate their interview at any time if they wished. All participants were also specifically asked for permission to record the session using a dictaphone.

### Analyses

Inductive thematic analysis techniques were employed in the coding and analysis of the data gathered. Data driven analysis strategies involve detailed readings of the raw data, from which concepts, themes, or models are derived based on the interpretation of those analysing the data [16]. This approach was best suited to the aim of the groups and interviews, which was to gather descriptive exploratory data concerning perceptions of the first drafts of the iCST materials. The transcripts were examined in conjunction with the field notes, which were used to clarify any points recorded as ‘inaudible’ in the transcripts, and any comments, which required further contextual information in order to be understood. Excerpts of text were extracted from the transcripts and used as labels for categories emerging from the data (eg: ‘potential difficulties’). Two researchers analyzed the data independently, entering the categories and supporting quotes from the transcripts into a spreadsheet. They then compared results and collaboratively reviewed any excerpts that could be coded to more than one category to reach consensus over their category placement. Throughout the analysis, the categories were continually refined to identify the themes most relevant to our evaluation objectives. Data from the groups and interviews was collated, then examined further by source (carers and people with dementia) to identify any variations in views. No specialist software was used to perform the data analyses.

## Results

Of the total sample of 52 participants; 14 carers and 18 people with dementia took part in the focus groups, and 10 carers and 10 people with dementia participated in the interviews. Full demographic information is shown in Table 2.

**Table 2:** **Demographics of carers and people with dementia participating in focus groups and interviews**

**Characteristics**		**Focus groups (%)**	**Individual interviews (%)**
**People with dementia**		*n =* 18	*n =* 10
Gender	Female	11 (61)	5 (50)
Mean age (years)		80.50 (*SD =* 5.80)	84.44 (*SD =* 4.10)
Ethnicity	White	18 (100)	10 (100)
**Carer**		*n =* 14	*n =* 10
Gender	Female	8 (57)	7 (70)
Mean age (years)		65.23 (*SD =* 9.65)	67.67 (*SD =* 14.35)
Ethnicity	White	11 (79)	10 (100)
Relationship	Spouse	7 (50)	6 (60)
	Child (son/daughter)	7 (50)	4 (40)
Living status	Spouse living with person	6 (43)	6 (60)
	Adult child living with person	2 (14)	3 (30)
	Person lives alone	4 (29)	1 (10)
	Person lives in care home	2 (14)	0
Mean years caring		5.61 (range 1–16, *SD* = 4.03)	2.89 (range 1.5-7, *SD* = 1.78)

The following themes emerged from the thematic analysis of the focus groups and interviews: ‘effects of mentally stimulating activities’, ‘the range of mentally stimulating activities’, ‘feasibility of a home based programme of mental stimulation’, and ‘quality of the materials’.

### Theme 1: Effects of mentally stimulating activities

People with dementia emphasised the importance of mental stimulation citing benefits such as keeping up to date with everyday events, increasing sense of well-being, learning, improving the mind, and preventing cognitive deterioration.

*‘…save us going backwards this is an advance on anything that will help us talk and improve our thoughts….’* (Person with dementia: Focus group 1)

In the interviews, people with dementia spoke about mentally stimulating activities as a way of occupying their time in a meaningful way, linking being active to the ability to retain a sense of self.

*‘Can’t give you a proper reason but it gives you an activity, doesn’t it? There’s activity there, and without it you’re nothing.’* (Person with dementia: Interview10)

Carers noted several benefits of mentally stimulating activities including; better quality of life for the person, improvements in mood, helping the person to think back, and increasing their alertness. There was consensus that it didn’t matter whether the person could remember the activity they had done (and indeed, often they would forget soon afterwards) as long as they had enjoyed it and been stimulated for a little while.

*‘I mean, we go to the theatre, we come home, not even two minutes after we've left there, she doesn't remember we've ever been, but that buoyant feeling is good.’* (Family carer: Focus group 1)

Although people with dementia seemed to value mental stimulation, several carers said the person they were caring for did not seek out mentally stimulating activities independently, and those that had attempted to engage their relative in activities reported difficulty motivating them. Interestingly, dependence on the carer for stimulation was acknowledged in one of the groups for people with dementia.

*‘May I just say I believe that we are all crying out for help and stimulation but we can’t, haven’t so much got ideas in our own head as we hope other people can encourage us.’* (Person with dementia: Focus group 1)

### Theme 2: The range of mentally stimulating activities

Both carers and people with dementia suggested that quizzes stimulate the mind and can be educational. Puzzles (eg: crosswords, jigsaws) were also a popular suggested activity, along with games such as cards and dominoes. People with dementia said that reading the newspaper keeps the mind stimulated. However, a carer commented that activities with a visual or auditory element were more worthwhile than just sitting and reading.

Watching TV was mentioned by people with dementia as a way of keeping up to date. The notion of ‘keeping up to date’ was repeatedly discussed, which suggests it is perceived as a key function of mentally stimulating activities.

*‘I mean, watching the box…you…“Cor! No, I didn’t know that!” That goes round the world and keeps you more up to date with everyday happenings.’* (Person with dementia: Interview 4)

People with dementia highlighted the need to keep both the brain and the body active, citing activities such as dancing, keep fit classes, sports, and yoga as valuable sources of mental and physical stimulation. Some carers also identified physical activities such as gardening and bowling as forms of stimulation, but they focused largely on activities requiring no physical exertion.

### Theme 3: Feasibility of a home-based programme of mental stimulation

#### Delivering the programme at home

The idea of a programme of mentally stimulating activities was generally well received. Some carers said it would be particularly useful to have activities to do together in the winter when they might be isolated by bad weather. People with dementia said that they would like to do activities at home, but emphasized that they would need someone to help them.

*‘The idea of activities (in the home) is good, people with dementia just need assistance with it.’* (Person with dementia: Focus group 1)

A concern for people with dementia living alone was identifying who would be able help them with the programme. For those who were co-habiting or regularly visited by relatives, their worry was not who would do the activities with them, but whether anyone would have the time, especially if their carer had a job. Carers also expressed this concern. Some people with dementia felt that they were able to keep themselves busy at home without doing activities, and prioritized tasks they felt had to be done (eg: housework, cooking).

#### Potential difficulties in delivering the programme

Carers volunteered an array of anticipated difficulties with the programme contextualized within their own personal circumstances. Feeling burdened by caring responsibilities might reduce willingness to deliver the programme:

*‘This kind of programme that requires all that amount of patience on top of the patience that you have to exercise for the everyday care is a lot to ask of a carer.’* (Family carer: Focus group 2)

Perceiving the programme to be too demanding for both themselves and the person they are caring for might compromise capacity to complete the programme:

*‘You know, there's a physical side of it and a mental side of it, I don't know how many carers would be able to follow this programme consistently for 25 weeks.’* (Family carer: Focus group 2)

Carers anticipated difficulty engaging their relative in activities without encountering resistance from them:

*‘You know, he would expect me to do it, but at the same time when I'm doing it, he would ask me ‘why all these?’ you know, so I'd just have to say ‘well, it's to help you, you know, to remember things, he would say “enough is enough”.’* (Family carer: Focus group 2)

The length of the programme and adhering to a ‘formal’ structure might impact the success of sessions:

*‘I find it difficult to identify who actually would give the programme because I think anyone from the family, it probably wouldn't work because it's too formal…’* (Family carer: Focus group 2)

Further difficulties identified included; lack of time due to work or other commitments, the person’s level of cognitive functioning, and maintaining motivation to deliver the programme:

*‘Keeping the person delivering it is just as important as the person receiving it, in fact, more so.’* (Family carer: Interview 7)

Some carers suggested that the programme would be more successful if delivered by a professional (ie: therapist, day centre staff), or a paid carer. It was thought that a ‘stranger’ or ‘outsider’ might elicit more of a response from a person with dementia than a family member. Carers were concerned that their relative would be less co-operative with them, and this could potentially cause them or the person distress.

*‘I could probably do the job better with someone else but my own wife! I think you can be too close. I feel you should, you need to be detached a little bit, and I couldn’t be detached, bearing in mind, you know, the situation.’* (Family carer: Interview 5)

The view that the programme would be more suitable for delivery by a professional was not shared by all carers; many either did not comment on the involvement of a professional, or felt that they would be capable of delivering the programme themselves with training and support. Some carers saw scope for the involvement of other family members or friends in the programme, whilst others considered it a task they would undertake by themselves.

*‘I’m not saying it’s wrong to have a member of staff, but I think the person, like me and Eric, would do it quite nicely together.’* (Family carer: Interview 10)

*‘[…] it’s something, mum, you could join in with dad as well. Once you get the idea of what’s going on, I think it would be good for you.’* (Family carer: Interview 6)

The level of support appeared to influence how feasible carers considered the programme to be. Those with little support from family members tended to speak about barriers such as lack of time or feeling burdened.

#### Appropriateness of home based activities

A carer commented that their relative with dementia expects to take part in stimulating activities in settings like the day centre or clubs, but would not be interested in doing activities at home.

*‘This would probably be very appropriate in a more formal setting like at a day centre […] with my mother, she recognizes that she is going to a day centre for activities and this could form part of that activity and she would accept that.’* (Family carer: Focus group 2)

Carers were aware that their relative might experience some trepidation about taking part in the activities at home, which might influence how receptive they were to the programme. However, it was suggested that any concerns could be overcome if the programme was presented in an appealing and relaxed way.

*‘(Sessions should be) more subtle, so no one feels testy. It’s more of a conversation and discussion rather than “it’s therapy time now”.’* (Family carer: Interview 7)

The tone of the activities was considered to be important as, if not pitched correctly, there might be a risk of the person viewing the activities as ‘childish’ or ‘boring’.

*‘Dad felt at first that it was going to be treating him like a child […..] but I think once it comes to doing the manual, he’ll realize it can be quite fun […] It mustn’t become a bore, a chore. It’s got to be fun. Dad’s got to enjoy it.’* (Family carer: Interview 6)

#### Duration and frequency of the sessions

The necessity of flexibility was discussed in relation to how many sessions could be completed per week, the duration of each session, and when sessions would take place. Most carers agreed that completing three sessions a week would be feasible, but perhaps not always possible depending on factors such as motivation (both the carer and person with dementia), mood, or needing to prioritize other tasks.

*‘I can imagine saying to him ‘come on we'll have a game of skittles’ and he'd say ‘oh I don't feel up to it at the moment’. There's all those factors to consider really so then, by the time you come to do it on that day, something else has gone on and it hasn't happened. So I think the flexibility here is important.’* (Family carer: Focus group 3)

In an interview one carer commented that they should not feel under pressure to complete three sessions per week as failing to reach this target might de-motivate them.

*‘If someone thinks, “oh god, I haven’t done 3!”, it’s like when you start off on evening classes. You’re really enthusiastic in the beginning and then, “I’m not really enjoying this”. […] I think you need to get something across, “well if you don’t do 3, it’s not the end of the world.’* (Family carer: Interview 7)

There was general agreement that spending 20–30 minutes on an activity would be possible, however many carers expressed a preference for short, informal sessions and suggested breaking down sessions across the day. Some carers pointed out that if sessions were any longer, they would be too tiring for the person with dementia. Incorporating rest breaks was suggested if the carer felt the person was bored or tired. By contrast, some carers were concerned that 20–30 minutes would not be a sufficient amount of time in which to complete the activities.

Carers expressed a preference for a more pragmatic approach to scheduling activities. They placed emphasis on having the freedom to do sessions when they felt like it, rather than setting specific times during which they must be completed. Carers’ perceptions of the session structure varied. Whilst some carers acknowledged the advantage of sessions being delivered in a consistent and structured way, others indicated they may not adhere to the structure outlined in the programme.

### Theme 4: Quality of the materials

The response to the first draft of the iCST manual and activity workbook was overwhelmingly positive. Carers felt that both manuals were clearly laid out and written in a way that was easy for them to understand. Many of the participants commented on how visually appealing they found the materials, notably the quality of the images used in the activity workbook, and the clear layout and professional look of the manual.

*‘I like the attractive cover. It gives one the impression it’s going to be interesting.’* (Family carer: Interview 2)

People with dementia indicated a preference for images rather than lengthy blocks of text. The clarity of the content of the manual was consistently highly rated, as was the selection of activities provided. Carers indicated that the tone of the language and terminology used were appropriate. All of the carers felt that the manual was easy to understand and the instructions clear enough to enable them to deliver the activities.

*‘Well it was plain speaking, it wasn’t fancy words […] It was straightforward so you couldn’t mess about you know, you wouldn’t make a mistake reading it would you? I found it good.’* (Family carer: Interview 4)

## Discussion

This study yielded valuable insight into the needs of service users for the iCST programme, and the importance of mental stimulation, both from the point of view of carers and people with dementia. Carers and people with dementia responded positively to the first drafts of the iCST manual and activity workbook, particularly the clarity of the language, range of ideas, and professional look of the materials. Feasibility issues, such as finding time to do the sessions, were identified and possible solutions offered by participants. This gave the research team an idea of the support carers will need in delivering the programme, as well as an understanding of likely reasons for non-adherence.

### Mentally stimulating activities

Carers and people with dementia emphasized the importance of being mentally active, attributing a wide range of cognitive, emotional, and functional benefits to taking part in mentally stimulating activities. This reflects the notion of ‘use it, or lose it’ proposed by Swaab [17]. The emotional impact of mentally stimulating activities was also highlighted. Carers felt that being mentally stimulated could improve quality of life and have a positive impact on mood. People with dementia placed emphasis on the need for meaningful activity in order to retain their sense of self, and provide continuity between ‘now’ and other stages in their life. These findings are consistent with those of Phinney, Chaudhury & O’Connor [18] who suggested that people garner meaning from involvement in activities in 3 ways: the pleasure and enjoyment of their experience, the feeling of belonging, and the ability to retain a sense of autonomy and identity. The significance of meaningful activity is also stressed by older adults without dementia [19]. However, the experience of dementia may mean that involvement in activity becomes more challenging. In particular ‘independent’ involvement, which was acknowledged by people with dementia and carers in this study, who noticed an increased reliance on others to provide opportunities and support in engaging in meaningful activities.

### Feasibility of delivering a programme of mental stimulation at home

Much of the data gathered from carers about the feasibility of the programme was focused on practical issues that might arise whilst delivering the activities. Largely, their receptiveness to delivering the programme appeared to be determined by whether practical issues were viewed as insurmountable barriers or difficulties that could be overcome.

### Time

Carers stressed the need for the programme to be flexible. Certainly, providing care often reduces the time available for other activities [20]. The impingement on time caused by occupying a caring role, and how well it is managed may lead to perceptions of role conflict and overload [21]. The recommended duration and frequency of iCST sessions was based on the intervention schedule of a home-based carer led programme of reality orientation evaluated by Onder *et al.* [22] and consultations with people with dementia and carers prior to the development of iCST. However, adherence to this intervention was not measured, thus it is difficult to use the study as a model to assess the feasibility of the proposed iCST intervention schedule. Further information about the feasibility of the proposed duration and frequency of sessions will be obtained from a field testing phase.

### Impact of Caregiver Burden (CB)

The perception of the feasibility of delivering the programme may be determined by the experience of CB. The functional level of the person with dementia, the extent of care provided, and the care-giving context have been identified as potential predictors of CB [20]. Consideration of the care-giving context and its impact on CB may reveal why a minority of carers felt the iCST programme was not feasible, and additionally, why several carers suggested it would be more suitable if delivered by a professional.

### Impact of family dynamics

Some carers were doubtful they would be able to engage their relative in a programme of activities at home. An understanding of the role relationship between the care-giver and the care recipient may provide insight into this belief. Pruchno, Burant & Peters [23] suggest that family histories influence the interactions between the carer and care recipient. The personalities of the carer and cared for can also impact these interactions [24]. The dyad develops expectations for the care-giving role, which define the basic parameters for the appropriateness of certain care tasks [20]. Delivery of a therapeutic intervention by a family member may not be deemed appropriate by the family member themselves, or their relative with dementia, or both based on their expectations. In this study the ‘appropriateness’ of a family carer delivering the programme was questioned by carers, but by contrast people with dementia welcomed the idea. It remains to be seen how the programme will be received by the dyad in practice and this is likely to depend largely on the context of the relationship, and perhaps the person with dementia’s understanding of the purpose of the programme.

### Skill base of the carer

Several studies have demonstrated that family carer led interventions are feasible and can yield positive outcomes for the carer including improvements in well being [25], and reduction in depressive symptoms [26], as well as improved cognition [22,25,26] for the person with dementia. These findings suggest that, contrary to the opinions expressed by some carers in the focus groups and interviews, interventions can be delivered by family members. With adequate training, accessible materials and a support system in place, it will be possible to equip carers with the skills they require to deliver the iCST intervention.

### Formal structure of sessions

Carers discussed the idea of adapting the session structure so that it would feel more ‘natural’, anticipating a formal session would not be appealing to their relative. Prospectively, this data indicates we may expect issues around intervention fidelity in the field-testing phase of the trial. Intervention fidelity can be defined as ‘the adherent and competent delivery of an intervention by the interventionist as set forth in the research plan’ [27]. Adopting the ‘Technology Model of Intervention Fidelity’ whereby the intervention package includes a manual, training, and incorporates regular monitoring of the interventionist [28] may increase the likelihood of carers implementing iCST as specified in the treatment protocol.

### Methodology strengths

Focus groups and interviews were selected as complementary methods of data collection to gather data with both depth and breadth [29]. An advantage of implementing a combination of methods, is that we were able to gather data from carers and people with dementia with a range of experiences efficiently, and supplement the emergent opinions and comments with in depth data gathered from the interviews [30]. Furthermore, it is thought that the use of a combination of qualitative methods can give more accurate and reliable response to research questions [31].

### Methodology limitations

A limitation of the focus group data gathered about iCST activities from people with dementia is that the activities were carried out in a setting bearing no resemblance to the intended intervention. However, an advantage of carrying out interviews is that the activities could be tested in a one-to-one capacity, which gave us a more representative insight into the quality and the appropriateness of the activities. As described earlier (see ‘methods’), three of each type of focus group (person with dementia, carer, and collaborative) were planned, however only six were carried out due to time constraints. At times the general outlook and perceptions of the carers participating in each of the focus groups were notably different. A further carer group may have been useful to moderate some of the more conflicting opinions expressed. However, when the data gathered from the 10 individual interviews was considered alongside the data from the focus groups, it appeared that many points were reiterated by multiple participants, indicating adequate data saturation.

Across all of the focus groups, at times it was difficult for the moderators to keep the carers and people with dementia ‘on topic’. In the people with dementia groups this may have occurred because focus group discussions rely on short-term memory and verbal communication, which are typically impaired [32]. Some carers saw the groups as an opportunity to share experiences or ‘complaints’ about their caring role in general. Experience of this issue was also reported by Qazi, Spector & Orrell [33], and appears to be a common limitation in qualitative methods involving service users. The problem of deviation from the questions in the topic guide also occurred in the interviews. Despite this, a substantial amount of informative data was gathered overall by means of both qualitative methods, so the impact of instances of lack of meaningful data was minimal. According to Morgan & Kreuger [34] and Morgan [35], the quality of data gathered can be attributed to factors such as choice of relevant questions, and appointment of qualified moderators in the data collection. Certainly in this case, the topic guide should have been more closely followed, and perhaps more experienced moderators selected to perform the interviews, as these errors compromised the data quality in some cases.

The groups could have been more ethnically diverse, with a majority of attendees being of a white ethnic background. The data was used to inform the development of the second draft of the manual, thus the activities included in the programme and materials included in the activity workbook may not have cross-cultural appeal. However, in line with work on group CST, it is likely that cultural adaptation would be needed for different groups.

### The development of iCST within the context of current research

Previous studies by Moniz-Cook *et al*. [25], Quayhagen & Quayhagen [26] and Onder *et al*. [22] have been influential in the development of the iCST programme. The intervention piloted by Moniz-Cook *et al.* [25] consisted of active training in memory management including, cognitive stimulation, orientation, and counselling with psycho-educative elements. Due to the multi-component nature of the intervention it is difficult to determine exactly how the cognitive stimulation element contributed to the positive outcomes reported. However, given that iCST is a well-defined programme cognitive stimulation alone, the impact of participation for people with dementia and carers will be more directly measurable. Although the intervention tested by Onder *et al.* [22] was structured and manualised, no formal measures of adherence were collected, thus the level of treatment fidelity could not be established. The potential risk of failure to adhere to the intervention as specified in the protocol is that any potential benefits of participation may be compromised or underestimated. In order to maximize treatment fidelity, plans to collect detailed adherence data at frequent intervals have been built into the design of the iCST trial to ensure the intervention is delivered as intended. Finally, the iCST trial will benefit from having a larger sample of participants than the previous studies identified.

## Conclusion

The proposed idea of an individualised, home based programme of CST and the sample materials presented were well received by both carers and people with dementia. The focus groups and interviews yielded valuable insight into the feasibility of the programme. Carers’ estimations of feasibility appeared to be shaped by their preconceptions about iCST and the anticipated experience of delivering an intervention. These preconceptions, especially those focusing on difficulties or negative outcomes, could create barriers in the delivery and effective implementation of the programme. The findings of the groups and interviews were valuable in that they identified these potential barriers at an early stage. The next phase in the trial will be to field test the programme prior to a large scale RCT in accordance with the guidelines outlined in the MRC Framework.

## Abbreviations

APA: American Psychiatric Association
CB: Caregiver burden
CST: Cognitive Stimulation Therapy
DSM-IV: Diagnostic and Statistical Manual of Mental Disorders (4^th^ Edition)
HTA: Health Technology Assessment
iCST: Individual Cognitive Stimulation Therapy
Maintenance CST: Maintenance Cognitive Stimulation Therapy
MRC: Medical Research Council
NELFT: North East London Foundation Trust
NHS: National Health Service
NICE: National Institute of Clinical Excellence
RCT: Randomised Controlled Trial
